# Ventro-Fixation of the Uterus

**Published:** 1894-10-20

**Authors:** 


### Medical Progress and Hospital Clinics.

[*The Editor will be glad to receive offers of co-operation and contributions from members of the profession. All letters should be addressed to* The Editor, The Lodge, Porchester Square, London, W.]

### VENTRO-FIXATION OF THE UTERUS.

The subject of displacements of the uterus has been the battlefield of many controversies, and even the one topic of retroflexion has a large and disputatious literature all its own. Time was when the causal relation between retroflexion and its symptoms was denied, and the mechanical treatment of the condition, even by such mild measures as the pessary and the sound, was looked on by many as useless if not worse. The proposal then to perform lapaotomy and to sew up the errant uterus to the abdominal wall, an operation which is steadily gaining ground in the special hospitals, must to some appear sufficiently startling to make a consideration of the position desirable, especially as one hospital has been publicly attacked on the ground of its mortality in regard to operations performed merely for the relief of pain and disability, as distinct from those undertaken for the saving of life. The operation of hysteropexy, or ventro-fixation of the uterus, has been done mainly for two conditions, viz., retroflexion and prolapse, or a combination of them; but it is in the first of these that it has seemed to give the best results, and to this we will chiefly direct our attention. It is no use now raising old controversies as to whether the symptoms of retroflexion are due to the mere bending of the womb, to its enlargement, to its congestion, or to the accompanying displacement of the ovaries; the facts have to be recognised that some women who are afflicted with this malposition, and who suffer such pain and discomfort on every attempt at exertion as to be practically debarred from work and usefulness, are restored to health and activity by well-devised local measures; that others, although temporarily relieved by the support given by pessaries, are quite unable to bear their continued use; and that others again are unable to get any relief whatever from treatment *per vaginam*, in consequence of some complication of the malposition.

We have then a series of cases having approximately the same symptoms, associated with the same distortion of the uterus, the treatment of which to quote the axiomatic formula of Hart and Barbour consists of 1. Replacement of the retroflexed uterus; 2. Retention of it in its normal position by suitable means; and yet differing greatly so far as regards their susceptibility to treatment by the older methods.

The question then is not one of introducing ventrofixation of the uterus as an ordinary treatment for ordinary retroflexion, but whether those cases for which but little can be done *per vaginam* are to be dealt with from above or left to fate.

Now in regard to this it is important to bear in mind that difficult cases of retroflexion are very often those in which the retroflexion is complicated by other conditions, the relief of which from below is next to impossible. A retroflexion produced by a strain in a woman only partially recovered from a confinement or miscarriage may at once give rise to typical symptoms, may be diagnosed and replaced within a few days, or even hours, and by dint of a short course of treatment directed to the support of the uterus and the promotion of its involution may leave the patient none the worse. This, however, is very different from the state of affairs in the difficult cases. Here we may have to do with a more or less chronic condition of inflammation. Not only may there be endometritis, but, with a history suggesting recurrent attacks of perimetritis, there may be physical signs of the uterus being bent on itself and tied down by old adhesions or by the retraction of old effusions. Under these circumstances not only may attempts at reduction cause great pain, but the withdrawal of the sound may be followed at once by a rebound of the uterus to its old position, or the tubes may be so tender, or the ovaries so displaced, that even if the uterus can be straightened it may be impossible to bring the pressure of a pessary to bear in such a way as to maintain the improvement. No doubt relief can be obtained by rest and various methods of local depletion, but cure does not take place, the malposition continues, slight causes of congestion bring back the old train of symptoms, and the patients remain more or less chronic invalids.

Now, for cases of this sort, the operation of ventrofixation offers not merely a means of holding up the uterus, but of removing the condition which held it down.

For details of the operation we may refer to the work of Dr. Pozzi, " Traité de Gynæcologie," Dr. Leith Napier in the *British Gynæcological Journal* for August, 1893, Dr. Leech in the *Medical Chronicle*, April, 1894, and Dr. Braithwaite in the *British Medical Journal*, May 19th, 1894. Shortly it is as follows: After all inflammatory complications have been subdued, the abdomen is opened as for oöphorectomy; the finger is introduced, the fundus sought for, any adhesions which may be discovered broken down, and the ovaries and tubes are freed, if tied down in abnormal positions, and brought up for inspection if necessary. The uterus is then seized by a small vulsella and drawn up, and its anterior surface is firmly sewn to the abdominal wall in the middle line.

The fit cases then for ventrofixation are those in which the uterus is acutely retroflexed, or retroflexed and prolapsed, but remains mobile, in which pessaries have been tried in vain for its support, and the patient is forced in consequence to lead an invalid life. Under such circumstances there is strong evidence that the operation is on the average a successful one, although not in all cases certainly curative; but a considerable number of cases have been reported in which it was so effectual that pregnancy afterwards took place and labour proceeded without untoward complications.

Yet there are dangers, and deaths have occurred; which brings one at once to the point that it is not to be looked on as a treatment for ordinary cases. It is not a substitute for the pessary, but comes in most usefully where this has failed. To realize the true meaning of the few deaths that have taken place one must bear in mind that the condition for which the operation is proposed is one of such gravity, at any rate one causing such misery, as to have led men to propose, and women to submit to, operative interference of a much more serious nature. Not only have all sorts of plastic operations been done on the vagina and its outlet, but the round ligaments have been shortened or fixed to the abdominal wall, attempts have been made to tear down posterior adhesions by the action of the finger within the dilated uterus, a whole series of operations has been devised for fixing the fundus in a better position by sutures through the vagina, Douglas' pouch has been opened and stuffed with gauze, alcohol has been injected to produce inflammatory retraction of the utero-sacral ligaments, &c. When considering the propriety of ventrofixation one must bear in mind the serious operations for which it is a substitute, and must not forget that its difficulties are not so much connected with the mere fixing of the uterus, as with the treatment of adhesions and conditions of ovaries and tubes which are quite out of reach of anything short of opening the peritoneum. We cannot doubt then that this operation will prove a valuable addition to the surgeon's means of dealing with a condition which makes life a constant martyrdom to many women; but to such cases it should at present be confined.

There is, however, at our disposal another method, and even a simpler one, for slinging up the uterus in cases of version or prolapse, which we owe to the suggestion of Dr. Keith—viz., the removal of one or other ovary, preferably the right, and the fixing of the pedicle outside by the clamp. The operation is extremely simple, an oöphorectomy in fact; but instead of the pedicle being tied it is fixed outside by the clamp, being drawn up sufficiently far to hold the uterus in the desired position. This operation is stated to have been performed in about fifty cases without any evil consequences, and with complete success in regard to the relief of the prolapse.

